# Age-related differences in limb fat-free mass and fat mass in healthy Chinese Adults

**DOI:** 10.1038/s41598-018-25447-z

**Published:** 2018-05-22

**Authors:** Mei Bai, Rui Wang, Linhao Zhu, Guixin Li, Dongya Yuan, Li Wang, Tianbo Jin

**Affiliations:** 1 Key Laboratory of Molecular Mechanism and Intervention Research for Plateau Diseases of Tibet Autonomous Region, School of Medicine, Xizang Minzu University, Xianyang, Shaanxi 712082 China; 2 Key Laboratory of High Altitude Environment and Genes Related to Diseases of Tibet Autonomous Region, School of Medicine, Xizang Minzu University, Xianyang, Shaanxi 712082 China; 3 Key Laboratory for Basic Life Science Research of Tibet Autonomous Region, School of Medicine, Xizang Minzu University, Xianyang, Shaanxi 712082 China; 4 Department of Medical and Education, Tangdu Hospital, Fourth Military Medical University, Xi’an, Shaanxi 710069 China; 5 Key Laboratory of Resource Biology and Biotechnology in Western China (Northwest University), Ministry of Education, Xi’an, Shaanxi 710069 China

## Abstract

Fat mass (FM) and fat-free mass (FFM) are important elements to evaluate nutritional status. The aims of this study were to establish reference values for FM and FFM of limbs, develop percentile distributions and assess age-related regional differences in body composition by multifrequency bioelectrical impedance analyzer (BIA) in healthy adults. A cross-sectional study was conducted on 3419 healthy subjects, 1595 men and 1824 women. Regional FM and FFM were measured by BIA. FM in men remained stable in both upper and lower limbs, with reference values (25–75th percentile) of 1–1.5 kg and 4.9–7.2 kg, respectively. Women’s leg FM remained stable with aging (reference values 6.2–7.9 kg), increasing in their arms (0.9–1.5 kg for youngest, 1.3–2.3 kg oldest). The reference values of upper limbs FFM were 5.3–6.2 kg in men and 3.3–3.9 kg in women. Lower limbs FFM decreased with age in both gender: the reference values were 19.5–23.3 kg (men) and 13.8–15.4 kg (women) for 18–30 age group, and 17.3–20 kg and 11.2–13.1 kg, respectively, for 60+ age group. These data provided reference values of FM and FFM in both limbs, enabling the identification of age and gender-related changes in limb composition in healthy Chinese subjects.

## Introduction

The study of body composition is increasingly considered as an important aspect for the evaluation of nutritional and metabolic status. As is well known, significant changes in body composition occur with aging and are associated with higher risk of morbidity and mortality^1–3^. Large absolute differences are known to exist between young and old subjects of similar body size in the individual compartments that compose the fat-free mass (FFM)^4^. In addition, the loss of FFM and relatively increased fat mass (FM) with aging has been documented in different clinical settings^5–7^ and may occur even in healthy elderly adults^8^.

The decline of FFM and the related loss of lean tissues occurred with advancing age in elderly men and women, and were associated with physical impairment, termed sarcopenia, even in independently living healthy subjects^9^. Moreover, lower levels of physical activity and higher levels of sedentary behavior may lead to the development of sarcopenia obesity also in minority youth^10^. In brief, the loss of FFM is closed related to adverse health events such as disability^11^, balance disorders and falls^8^, hospitalization, and mortality^12^. In contrast, an increased FM may result in overweight or obesity, which is a major public health concern. Obesity is most commonly caused by a combination of excessive food intake, lack of physical activity, and genetic susceptibility^13^ and is a leading preventable cause of death worldwide, with increasing rates in adults and children. Furthermore, previous studies have showed that obesity increased the likelihood of various diseases and conditions, particularly cardiovascular disease, type 2 diabetes, respiratory problems, certain types of cancer and mortality, especially in adult populations^14,15^. In a word, either the loss of FFM or the gain of FM does harmful to health.

Majorities of cross-sectional and intervention studies have demonstrated the relationships of trunk fat, and limbs fat to cardiovascular disease, insulin resistance, blood lipids and inflammation. However, age and sex-dependent differences in limb FM and FFM in healthy subjects have not been clearly delineated, and results are not consistent across studies. The purposes of our study were to provide reference values for FM and FFM of the lower and upper limbs in Chinese adults, and to investigate regional changes occurring with aging by the latest multifrequency bioelectrical impedance analyzer (BIA) technology.

## Results

Tables 1 and 2 show the mean values for the general anthropometric features and the regional body composition characteristics of the sample between 18 and 82 y of age by age group, in men and women respectively. The mean height of subjects was 171.88 ± 6.06 cm in men and 159.78 ± 5.39 in women. Height was significantly lower in subjects older than 60 compared to the youngest in both men and women. The mean weight was 73.92 ± 10.69 in men and 57.68 ± 8.27 in women. Weight was highest in 31 to 40-y-old men and was lower in older age groups, however, it slightly increased with aging in women. BMI was peaked in oldest group for women, while remaining stable in men. Moreover, the prevalence of underweight (BMI < 18.5 kg/m^2^), overweight (BMI: 25.0–29.9 kg/m^2^) and obesity (BMI ≥ 30 kg/m^2^) were 1.50%, 43.82% and 6.21% in males, and 6.74%, 18.91% and 2.25% in females, respectively (data were not shown).

**Table 1:** General anthropometric and body composition characteristics (mean ± SD) by age group in men.

Age(year)	18–82	18–30	31–40	41–50	51–60	60+
n	1595	506	419	361	220	89
Height(cm)	171.88 ± 6.06	173.45 ± 5.76	172.42 ± 5.97	170.95 ± 5.99	170.11 ± 5.94	168.58 ± 5.68*
weight(kg)	73.92 ± 10.69	72.36 ± 11.71	75.69 ± 10.94	74.60 ± 9.58	73.78 ± 9.71	71.98 ± 8.50
BMI(kg/m2)	24.99 ± 3.16	24.02 ± 3.46	25.42 ± 3.15	25.50 ± 2.76	25.47 ± 2.78	25.32 ± 2.61
FM upper (kg)	1.24 ± 0.42	1.13 ± 0.45	1.28 ± 0.42	1.27 ± 0.38	1.33 ± 0.41	1.37 ± 0.37
FM lower (kg)	6.19 ± 1.90	5.91 ± 2.11	6.49 ± 1.93	6.26 ± 1.65	6.21 ± 1.72	6.04 ± 1.58
PBF upper (%)	17.32 ± 4.08	16.00 ± 4.50	17.57 ± 3.82	17.65 ± 3.48	18.41 ± 3.67*	19.54 ± 3.77*
PBF lower (%)	22.45 ± 3.98	20.95 ± 4.40	22.78 ± 3.75	23.09 ± 3.35	23.60 ± 3.52	23.99 ± 3.51
FFM upper (kg)	5.79 ± 0.71	5.74 ± 0.71	5.85 ± 0.73	5.84 ± 0.70	5.77 ± 0.69	5.58 ± 0.63
FFM lower (kg)	20.90 ± 2.73	21.59 ± 2.76	21.49 ± 2.76	20.47 ± 2.36	19.73 ± 2.46	18.81 ± 2.14
LMM upper (kg)	5.39 ± 0.70	5.35 ± 0.70	5.46 ± 0.72	5.44 ± 0.69	5.38 ± 0.68	5.19 ± 0.62
LMM lower (kg)	19.75 ± 2.59	20.40 ± 2.61	20.31 ± 2.62	19.35 ± 2.24	18.63 ± 2.33	17.77 ± 2.04

All data are presented as mean ± SD. *Significantly different from age group 18–30.

Abbreviations: BMI, body mass index; FM, fat mass; PBF, percent body fat; FFM, fat-free mass; LMM, lean muscle mass.

**Table 2:** General anthropometric and body composition characteristics (mean ± SD) by age group in women.

Age(year)	18–82	18–30	31–40	41–50	51–60	60+
n	1824	683	427	383	242	89
Height(cm)	159.78 ± 5.39	161.04 ± 5.35	160.18 ± 4.87	158.82 ± 5.32	158.36 ± 5.29	156.22 ± 5.47*
weight(kg)	57.68 ± 8.27	55.42 ± 8.25	57.65 ± 8.05	59.76 ± 7.98	60.17 ± 7.28	59.51 ± 8.60
BMI(kg/m2)	22.60 ± 3.11	21.36 ± 2.93	22.46 ± 2.92	23.67 ± 2.78	24.01 ± 2.80	24.41 ± 3.45
FM upper (kg)	1.49 ± 0.65	1.28 ± 0.60	1.44 ± 0.60	1.66 ± 0.62	1.73 ± 0.61	1.89 ± 0.77
FM lower (kg)	7.16 ± 1.52	7.01 ± 1.60	7.17 ± 1.49	7.36 ± 1.44	7.25 ± 1.38	7.15 ± 1.64
PBF upper (%)	27.95 ± 6.38	25.73 ± 6.24	27.44 ± 5.86	29.61 ± 5.47	30.74 ± 5.99	32.77 ± 7.21
PBF lower (%)	33.54 ± 3.78	32.04 ± 3.76	33.34 ± 3.27	34.54 ± 3.15	35.46 ± 3.41	36.63 ± 4.26
FFM upper (kg)	3.64 ± 0.48	3.51 ± 0.45	3.64 ± 0.46	3.78 ± 0.52	3.77 ± 0.44	3.67 ± 0.49
FFM lower (kg)	14.04 ± 1.46	14.67 ± 1.30	14.16 ± 1.26	13.81 ± 1.34	13.05 ± 1.20	12.18 ± 1.39
LMM upper (kg)	3.43 ± 0.46	3.30 ± 0.44	3.43 ± 0.45	3.57 ± 0.48	3.56 ± 0.42	3.46 ± 0.47
LMM lower (kg)	13.22 ± 1.35	13.80 ± 1.20	13.34 ± 1.17	13.01 ± 1.24	12.31 ± 1.11	11.50 ± 1.29

All data are presented as mean ± SD. *Significantly different from age group 18–30.

Abbreviations: BMI, body mass index; FM, fat mass; PBF, percent body fat; FFM, fat-free mass; LMM, lean muscle mass.

In men, the mean FM and PBF of the upper limbs were significantly higher in age group 60+ compared with youth (from 1.13 ± 0.45 to 1.37 ± 0.37 kg for FM; from 16.00 ± 4.50 to 19.54 ± 3.77 kg for PBF). The FFM and LMM tended to decrease in lower limbs; significant differences were only seen from 60 years old onwards. In women, the mean FM and PBF regionally increased in upper limbs and trunk but not in lower limbs. In addition, FM and PBF in women were constantly higher than in men. FFM and LMM tended to decrease in lower limbs, but not for upper limbs and trunk. In contrast to the FM and PBF, FFM and LMM were higher in men.

**Figure 1:**
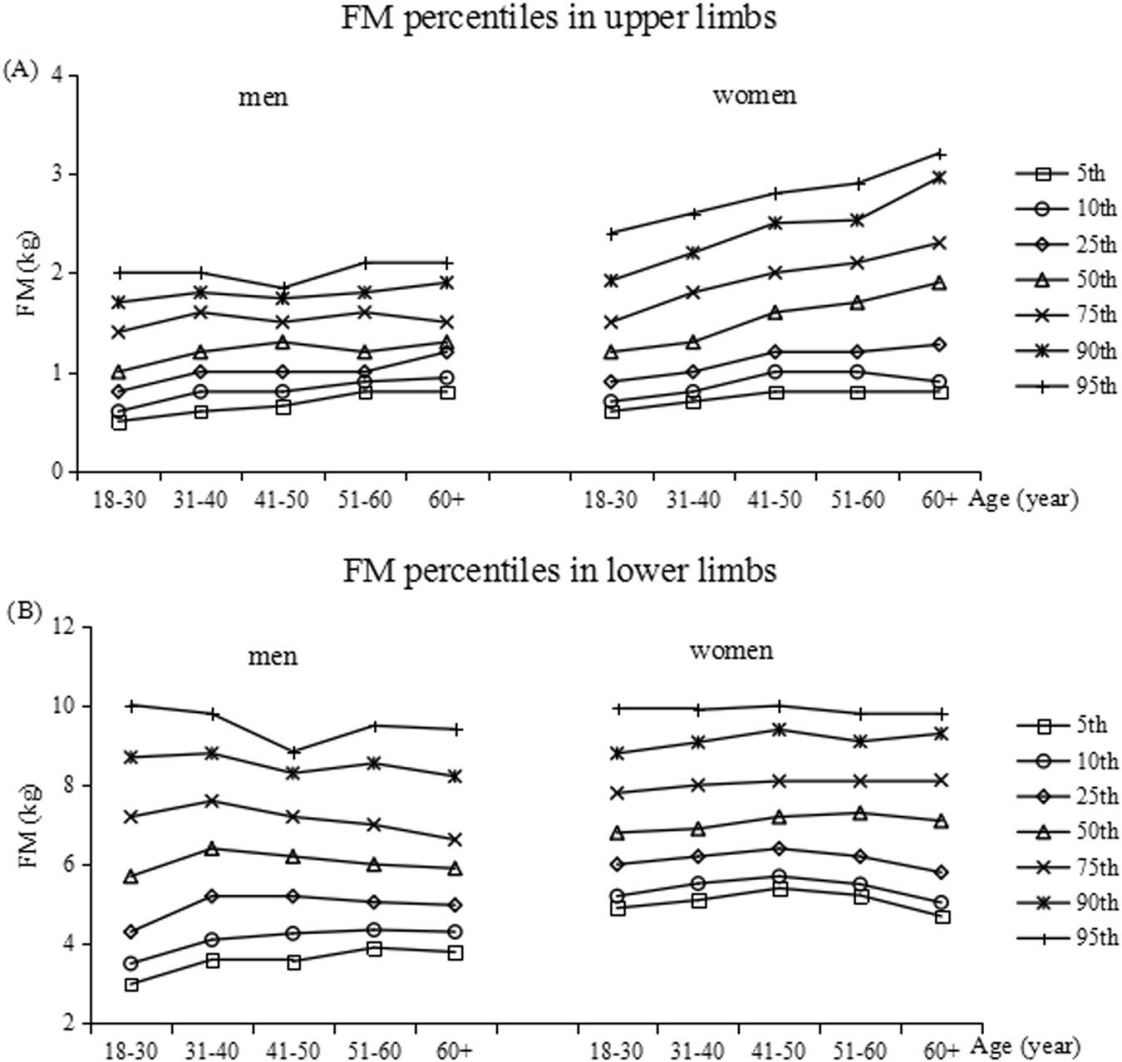
The percentiles for the distribution of FM values for the upper and lower limbs, in both genders. The left curves and right curves are for men and women, respectively.

**Figure 2:**
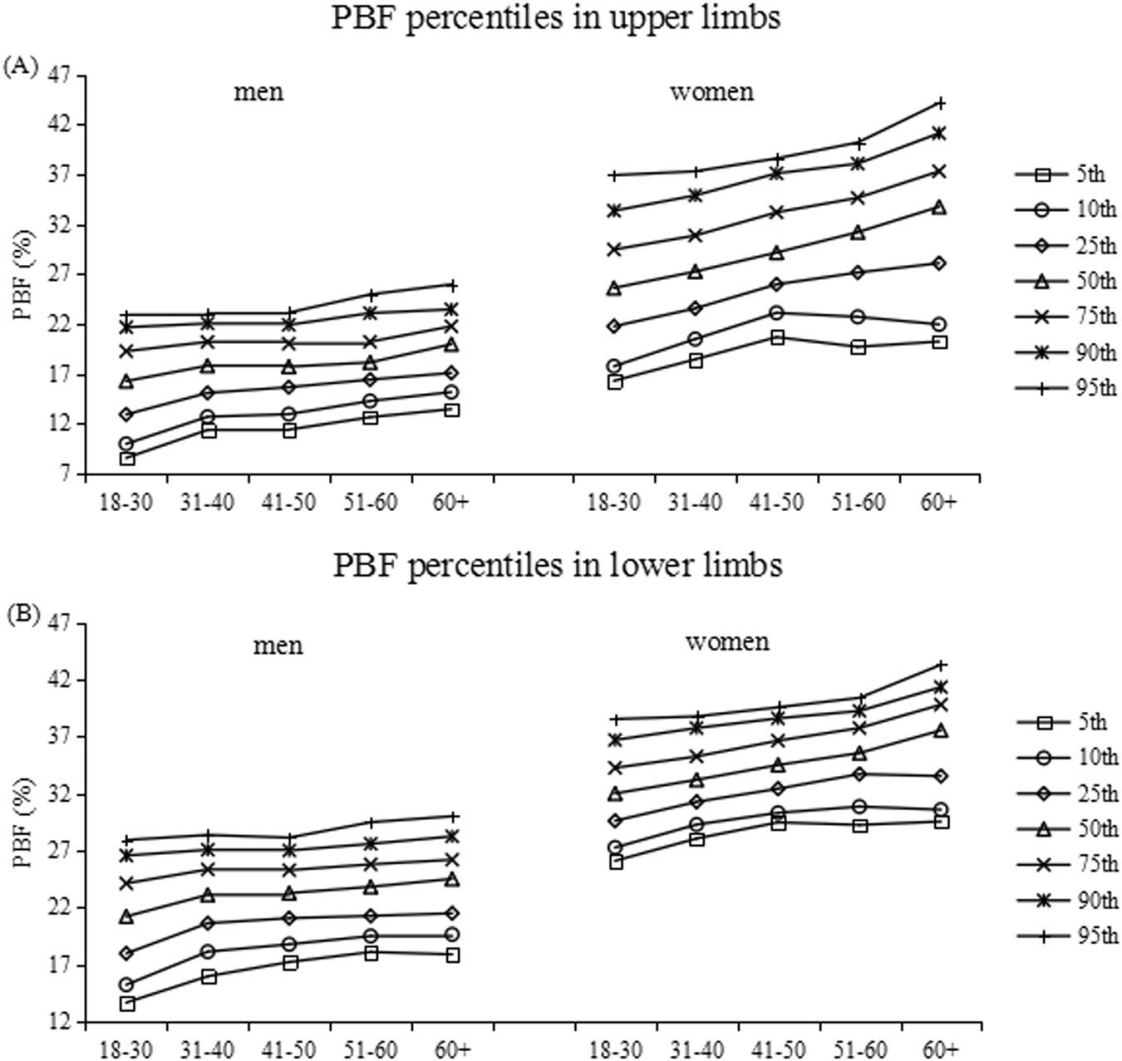
The percentiles for the distribution of PBF values for the upper and lower limbs, in both genders. The left curves and right curves are for men and women, respectively.

**Figure 3:**
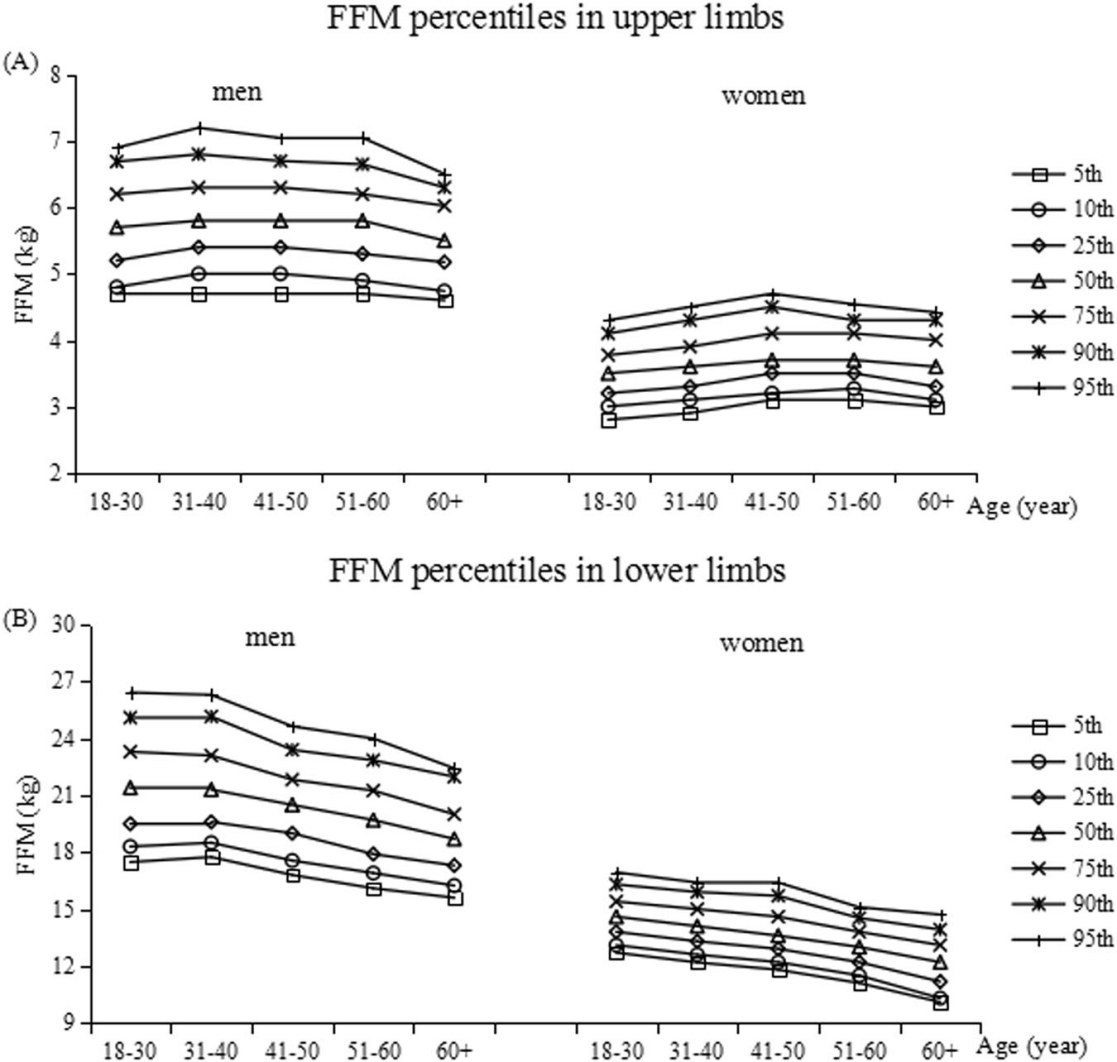
The percentiles for the distribution of FFM values for the upper and lower limbs, in both genders. The left curves and right curves are for men and women, respectively.

Tables 3–6 show the percentiles of the FM (kg) and FFM (kg) in the upper and lower limbs in both men and women. Each table is divided into age- and gender-specific percentile distributions (5th, 10th, 25th, 50th, 75th, 90th, and 95th). Figures 1–3 show the trend of the FM, PBF and FFM percentiles for limbs by age group in the two genders.

**Table 3:** Percentiles for limb fat mass in kg by age groups in men.

Age(year)	n	5th	10th	25th	50th	75th	90th	95th
upper limbs								
18+	1595	0.6	0.8	1	1.2	1.5	1.8	2
18–30	506	0.5	0.6	0.8	1	1.4	1.7	2
31–40	419	0.6	0.8	1	1.2	1.6	1.8	2
41–50	361	0.66	0.8	1	1.3	1.5	1.74	1.85
51–60	220	0.8	0.9	1	1.2	1.6	1.8	2.1
60+	89	0.8	0.94	1.2	1.3	1.5	1.9	2.11
lower limbs								
18+	1595	3.3	3.9	4.9	6	7.2	8.6	9.6
18–30	506	2.98	3.5	4.3	5.7	7.2	8.7	10.02
31–40	419	3.6	4.1	5.2	6.4	7.6	8.8	9.8
41–50	361	3.56	4.26	5.2	6.2	7.2	8.3	8.85
51–60	220	3.9	4.35	5.05	6	7	8.55	9.5
60+	89	3.79	4.3	4.98	5.9	6.63	8.22	9.41

**Table 4:** Percentiles for limb fat mass in kg by age groups in women.

Age(year)	n	5th	10th	25th	50th	75th	90th	95th
upper limbs								
18+	1824	0.7	0.8	1	1.4	1.8	2.3	2.7
18–30	683	0.6	0.7	0.9	1.2	1.5	1.92	2.4
31–40	427	0.7	0.8	1	1.3	1.8	2.2	2.6
41–50	383	0.8	1	1.2	1.6	2	2.5	2.8
51–60	242	0.8	1	1.2	1.7	2.1	2.53	2.9
60+	89	0.8	0.9	1.28	1.9	2.3	2.96	3.21
lower limbs								
18+	1824	5	5.4	6.1	7	7.9	9.1	9.9
18–30	683	4.9	5.2	6	6.8	7.8	8.8	9.94
31–40	427	5.1	5.52	6.2	6.9	8	9.08	9.9
41–50	383	5.4	5.7	6.4	7.2	8.1	9.4	10
51–60	242	5.22	5.5	6.2	7.3	8.1	9.1	9.8
60+	89	4.7	5.04	5.8	7.1	8.13	9.3	9.83

**Table 5:** Percentiles for limb fat-free mass in kg by age groups in men.

Age(year)	n	5th	10th	25th	50th	75th	90th	95th
upper limbs								
18+	1595	4.7	4.9	5.3	5.8	6.2	6.7	7
18–30	506	4.7	4.8	5.2	5.7	6.2	6.69	6.9
31–40	419	4.7	5	5.4	5.8	6.3	6.8	7.2
41–50	361	4.7	5	5.4	5.8	6.3	6.7	7.05
51–60	220	4.7	4.9	5.3	5.8	6.2	6.65	7.05
60+	89	4.6	4.74	5.18	5.5	6.03	6.3	6.51
lower limbs								
18+	1595	16.9	17.6	19	20.7	22.5	24.5	25.8
18–30	506	17.48	18.3	19.5	21.4	23.3	25.1	26.42
31–40	419	17.75	18.5	19.6	21.3	23.1	25.16	26.3
41–50	361	16.8	17.56	19	20.5	21.83	23.4	24.65
51–60	220	16.1	16.9	17.9	19.7	21.25	22.85	24
60+	89	15.6	16.24	17.3	18.7	20	21.98	22.43

**Table 6:** Percentiles for limb fat-free mass in kg by age groups in women.

Age(year)	n	5th	10th	25th	50th	75th	90th	95th
upper limbs								
18+	1824	2.9	3.1	3.3	3.6	3.9	4.3	4.5
18–30	683	2.8	3	3.2	3.5	3.78	4.1	4.3
31–40	427	2.9	3.1	3.3	3.6	3.9	4.3	4.5
41–50	383	3.1	3.2	3.5	3.7	4.1	4.5	4.7
51–60	242	3.1	3.27	3.5	3.7	4.1	4.3	4.54
60+	89	3.0	3.1	3.3	3.6	4	4.3	4.42
lower limbs								
18+	1824	11.77	12.2	13.1	14	15	15.9	16.5
18–30	683	12.7	13.1	13.8	14.6	15.4	16.3	16.94
31–40	427	12.19	12.6	13.3	14.1	15	15.9	16.4
41–50	383	11.8	12.2	12.9	13.6	14.6	15.7	16.4
51–60	242	11.1	11.5	12.2	13	13.8	14.53	15.1
60+	89	10.1	10.3	11.18	12.2	13.1	13.92	14.71

In both gender, FM remained stable in both upper and lower limbs, although this was not for upper limbs of women. The reference values (25–75th percentile) were 1–1.5 kg for upper limbs and 4.9–7.2 kg for lower limbs and were much the same in all age groups in men. In women, the 25th to 75th percentile values of upper limbs were about 0.9–1.5 kg in the youngest age group and 1.3–2.3 kg in the oldest age group, while 6.2–7.9 kg for lower limbs in all age groups.

Figure 2 shows graphs of the percentiles for the distribution of PBF values for the upper and lower limbs, in both genders. The PBF percentiles were higher in women than in men and increased in all percentiles across age groups. The 25th to 75th percentile range of upper limbs was about 12.9–19.3% in the 18 to 30 year-old age bracket and 17.1–21.8% in the oldest group, while 17.95–24.1% for the youngest and 21.5–26.2% for the oldest for lower limbs in men. In women, PBF of both legs and arms increased with age for the 25th to 95th, while this trend were not apparent in the 5th and 10th after 40 years old. The reference values of upper limbs were 21.8–19.5% and 28.1–37.3% for 18–30 years group and 60+ group, respectively. The reference values of legs for the 18–30 age group were 29.6–34.3%, while for the 60+ age group they were 33.5–39.8%.

The FFM percentiles were lower in women than in men and relatively more stable with aging for upper limbs in both men and women (Fig. 3). The 25th to 75th percentile range of arms for all subjects was 5.3–6.2 kg in men (Tables 5) and 3.3–3.9 in women (Table 6) and was much the same in all age groups. The FFM of lower limbs decreased in all percentiles with age in both sexes. The 25th to 75th percentile range was about 19.5–23.3 kg for the 18–30 year-olds and 17.3–20 kg for the 60+ year-olds in men, while 13.8–15.4 kg for the youngest and 11.2–13.1 kg for the oldest in women.

## Discussion

Body composition evaluation should be integrated into routine clinical practice for the initial assessment and sequential follow-up of nutritional status^16^. Our research provided FM, FFM and PBF percentiles distributed by age and sex-related suitable for use as normal reference values in nutritional assessments and clinical practice. A few significant differences of the parameters were demonstrated among age groups in both men and women.

Analyzing the percentile distribution curves, the FM of either lower or upper limbs showed no significant age-related differences in both men and women. However, PBF tended to increase slightly with age in both genders. This finding may support the results of previous studies, which found that appendicular FM peaked in age group of 60 to 74 years old in healthy adults^17^, and a progressive increase of FM% was also observed in patients from 20 to 80 years old^18^. In addition, a study by Toomey *et al*.^19^ demonstrated that the increased in body weight with aging may due to an increase in FM, not only in the trunk, but in the limbs as well^18^. It has been reported that loss of FFM and muscle mass are associated with aging^20^; our results consented with this. The older adults studied here lost FFM and muscle mass (data were not shown) with age even though they were healthy. The differences in age-related changes in regional body composition between men and women may affect the sex-based differences in age-related changes in health status.

Regarding limb composition, our study focused on gender-specific differences between upper and lower limbs in the various age groups. Confirming previously reported findings, this study showed that both FM and PBF at the upper and lower limbs increased considerably with advancing age in both men and women, but women seemed to maintain a more stable FM for legs. In addition to progressive increases in fat mass with age, progressive reduction in fat-free mass (FFM) is also noted. The phenomenon may associate with increasing sedentary lifestyle. In adults, several studies have suggested that FM in the lower limbs seems to have a protective effect against insulin resistance and dyslipidemia^21,22^ beyond total fat mass and trunk fat as assessed by dual energy x-ray absorptiometry (DXA)^20^. Indeed, some studies have found leg fat mass was an important determinant of cardiometabolic risk after menopause, reducing risk of hyperinsulinemia and insulin resistance, whereas trunk and arms fat was unfavorably associated with increased risk of cardiometabolic health^23^. These results suggest that, for a given magnitude of central or whole body adiposity, a larger proportion of leg fat may have a protective effect on cardiometabolic health^24^.

This study has some limitations. First, the relatively low number of people old than 60 seems to be an important limitation for the reference values of body composition. In addition, this study is a cross-sectional study, in which the healthy youths may not be healthy in old age. On the other hand, overweight and obesity subjects were regarded as healthy as well; thus, this point deserves special consideration.

In conclusion, the present data provided the reference values of FM and FFM in limbs, enabling the identification of physiological or pathological changes in limb composition for Chinese populations living in the Northwest and future investigation on pathological human conditions and differences between countries.

## Methods

### Ethics committee statement

This cross-sectional study was performed in compliance with the principles of the Declaration of Helsinki of the World Medical Association and obtained the permission from the Ethics Committee of Xizang Minzu University and Northwest University. All of the participants were informed of the cross-sectional study, and informed consent was taken from each participant.

### Study design and participants

This cross-sectional study was conducted on a sample of apparently healthy Chinese Han adults from Shaanxi Province. All subjects were volunteers, between 18–82 years old, who were recruited by a health management center. Informed consent was taken from each participant. All subjects were living independently and had no known pathologies or physical handicaps. The inclusion criteria were fitness for blood donation. Subjects with acute diseases, severe liver, heart or kidney dysfunctions cancer or other conditions capable of altering body composition were not recruited. The use of certain drugs (steroids, diuretics) was also a reason for exclusion. Furthermore, pregnant women and subjects with surgical hardware, implantable devices were excluded from the study. In the end, 3419 subjects were considered, 1595 of them men and 1824 women.

### Assessment of body composition

Body weight was measured to the nearest 0.1 kg using precision scales (Seca 711, Seca GmBH & Co Kg, Germany) with subjects wearing light clothing and no shoes; height was measured without shoes and recorded to the nearest millimeter using a stadiometer (Seca 711, Seca GmBH & Co Kg, Germany).

Body composition were calculated by a multifrequency bioelectrical impedance analyzer (BIA) (MC-980A; Tanita, Tokyo, Japan). The limb composition given by the machine’s software was used for this analysis. The participants were required to fast and avoid vigorous exercise for at least 2 h before BIA assessment. The BIA assessment was performed between 10:00 AM and 4:00 PM. The measurements were recorded by well-trained staff and completed within 30 s.

### Statistical analysis

The data obtained from the health management center were stratified into five groups according to age: 18 to 30 years, 31 to 40 years, 41 to 50 years, 51 to 60 years, and older than 60 years. All statistical analyses were performed with SPSS 15.0, and the level of significance was set at p = 0.05. Descriptive characteristics are expressed as mean standard ± deviation (SD). Significance was tested using the chi-square test and ANOVA. Age- and sex-specific percentile distributions of upper and lower limbs were calculated for FFM, FM, and PBF.
